# Cognitive effects of adaptive deep brain stimulation in Parkinson’s disease: stability without risk

**DOI:** 10.1186/s40001-025-03064-7

**Published:** 2025-08-29

**Authors:** Roberta Ferrucci, Fabiana Ruggiero, Edoardo Nicolò Aiello, Sara Marceglia, Marco Prenassi, Barbara Poletti, Francesca Cortese, Tommaso Bocci, Denise Mellace, Mattia Arlotti, Natale Maiorana, Francesca Mameli, Linda Borellini, Filippo Cogiamanian, Enrico Mailland, Nicola Ticozzi, Maurizio Vergari, Elena Pirola, Antonella Ampollini, Luigi Remore, Marco Locatelli, Sergio Barbieri, Alberto Priori

**Affiliations:** 1 Dipartimento di Neuroscienze e Salute Mentale, Fondazione IRCCS Ca’ Granda Ospedale Maggiore Policlinico, via F. Sforza 35, 20122 Milano, Italy; 2 Dipartimento di Oncologia ed Emato Oncologia, Università degli Studi di Milano, Via Santa Sofia 9/1, 20122 Milano, Italy; 3 Dipartimento di Neurologia e Laboratorio di Neuroscienze, IRCCS Istituto Auxologico Italiano, Piazzale Brescia 20, 20149 Milano, Italy; 4 Dipartimento di Ingegneria e Architettura, Università degli Studi di Trieste, Via Valerio 10, 34127 Trieste, Italy; 5 Presidio Ospedaliero San Filippo Neri, Unità di Neurologia, Via G. Martinotti 20, 00135 Roma, Italy; 6 ASST-Santi Paolo e Carlo, SC di Neurologia I, Via A. di Rudinì 8, 20142 Milano, Italy; 7 Dipartimento di Scienze della Salute, CRC Aldo Ravelli, Università degli Studi di Milano, Via A. di Rudinì 8, 20142 Milano, Italy; 8 Newronika S.p.A, Via T. Tasso 1, Cologno Monzese, Italy 20093; 9 Dipartimento di Fisiopatologia Medico-Chirurgica e dei Trapianti, Università degli Studi di Milano, Via F. Sforza 35, 20122 Milano, Italy

**Keywords:** Adaptive DBS, Continuous DBS, Memory, Reaction times, Language

## Abstract

**Background:**

Adaptive deep brain stimulation (aDBS) is a closed-loop system that adjusts stimulation based on patient biomarkers. This study evaluated the cognitive safety of aDBS in Parkinson’s disease (PD).

**Methods:**

Sixteen PD patients with bilateral subthalamic DBS underwent cognitive assessments (attention, language, memory) 6 days post-surgery during an 8 h protocol. Testing occurred at five time points: T1 (aDBS, medication “off”), T2/T4 (aDBS, medication “on”), and T3/T5 (aDBS “on”, medication “off”). Four patients followed the same protocol with continuousDBS (cDBS).

**Results:**

Results showed no cognitive fluctuations in aDBS patients (*p* ≥ 0.110). However, cDBS patients exhibited significant reaction time (RT) variations (*p* = 0.019), with RTs lower at T1 than T3 (*p* = 0.011) and T5 (*p* = 0.021), and at T4 compared to T2 (*p* = 0.002).

**Conclusion:**

These findings suggest that 8 h aDBS may not adversely affect cognitive performance, providing preliminary evidence of its cognitive safety and stability in PD.

## Introduction

Deep brain stimulation (DBS) of the subthalamic nucleus (STN) has become the treatment of choice for motor symptoms in late-stage Parkinson’s disease (PD) patients, with several studies also reporting post-surgery cognitive deficits of specific domains [1]. Longitudinal studies have reported beneficial effects of STN-DBS for up to 3 years [2], and studies that followed patients for 10 years argued that the benefits of STN-DBS might diminish over time, particularly for axial and non-dopaminergic symptoms [3]. However, other studies have shown that certain motor benefits, such as tremor reduction and improvement in levodopa-induced complications, may remain stable in the long term [4, 5]. Current DBS systems are open loop: the neurostimulator cannot capture the neuronal signals from the brain interacting with the deep brain electrode(s) [6]. Continuous (cDBS) systems deliver a continuous regular train of electrical pulses of fixed frequency, amplitude, and pulse width. Stimulation cannot be automatically adjusted to the patients’ needs at any given time, based on the symptoms, individual activity level, or medication cycle. Recent developments of the DBS consist in the adaptation of the impulses delivered by the DBS to the tissue in a closed-loop control (adaptive DBS; aDBS) [7].

Findings on the cognitive side effects of cDBS are highly heterogeneous [1]. Many studies have reported domain–/function-specific declines in language, memory, and executive function after cDBS implantation [8]. Other studies failed to report cognitive changes after surgery or when comparing “on” and “off” states of stimulation [8]. Such discrepancies might be due to variations in electrode placement, frequency of the stimulation, and total amount of energy delivered (TEED) to the STN [9, 10]. Recent evidence suggests that cognitive side effects may result, at least in part, from current spread to non-motor territories of the STN, which are functionally connected to cognitive networks [11, 12]. In this context, adaptive DBS (aDBS)—which delivers stimulation only in response to pathological neural activity—could represent a more physiological approach, potentially reducing off-target effects and preserving cognitive function [13].

Adjusting the amount of current delivered and limiting unnecessary activation of non-motor circuits could, thus, reduce the side effects of DBS systems on cognition [9, 13, 14]. Taken together, such findings suggest that aDBS systems might affect cognitive functions to a lesser extent than cDBS ones [13]. However, to the best of our knowledge, no studies to date have explored the cognitive safety of aDBS systems, while promising data on the safety, tolerability, and effectiveness of aDBS on motor disturbances have been recently reported [15, 16].

Given the above premises, the aim of the present study was to exploratively assess whether aDBS systems to the STN are cognitively safe in a cohort of PD patients.

## Materials and methods

### Participants

We enrolled 16 patients (mean ± SD; age: 57.56 ± 8.39; education 12.1 ± 4.0; UPDRS III: 29.6 ± 8.7; disease duration 9.9 ± 3.2 years; Hoehn and Yahr stage from 2.5 to 4; 12 males) with advanced PD who underwent a surgical procedure for bilateral subthalamic DBS implantation. All the clinical details of the patients are reported in Table 1. None of the patients experienced surgical complications. The study was approved by the institutional review board (Ethics Committee Milan Area 2) and conformed with the Declaration of Helsinki, and all patients provided written informed consent to the experimental procedures.

**Table 1.** Patient’s demographic clinical characteristics

Participant	Sex	Age at surgery	Education (years)	Disease duration (years)	MoCA score	Preoperative LEDD (mg)	Preoperative UPDRS III	Onset side
1	M	59	18	10	30	208	25	R
2	M	62	8	9	28,11	685,55	41	R
3	M	66	13	12	25,98	1494	25	R
4	M	49	8	8	28,28	1055	32	R
5	M	66	8	12	29,11	1228	22	L
6	M	46	13	7	27,15	//	22	R
7	M	47	13	11	30	1080	19	R
8	M	58	13	7	27,52	1856,66	35	R
9	F	67	11	16	25,98	1648	23	R
10	M	70	8	12	24,72	872	34	L
11	F	46	8	6	26,28	1436,1	27	L
12	M	66	8	3	27,11	1436,1	17	L
13	M	59	17	14	25,52	2248	//	L
14	M	52	18	12	23,52	935	45	L
15	F	58	18	10	24,52	818	40	R
16	F	48	11	9	22,15	793	37	R

MoCa: Montreal Cognitive Assessment; LEDD: levodopa equivalent daily dose; UPDRS: Unified Parkinson’s Disease Rating Scale; L: left; R: right; //: missing data

### Procedures

**Fig. 1.**
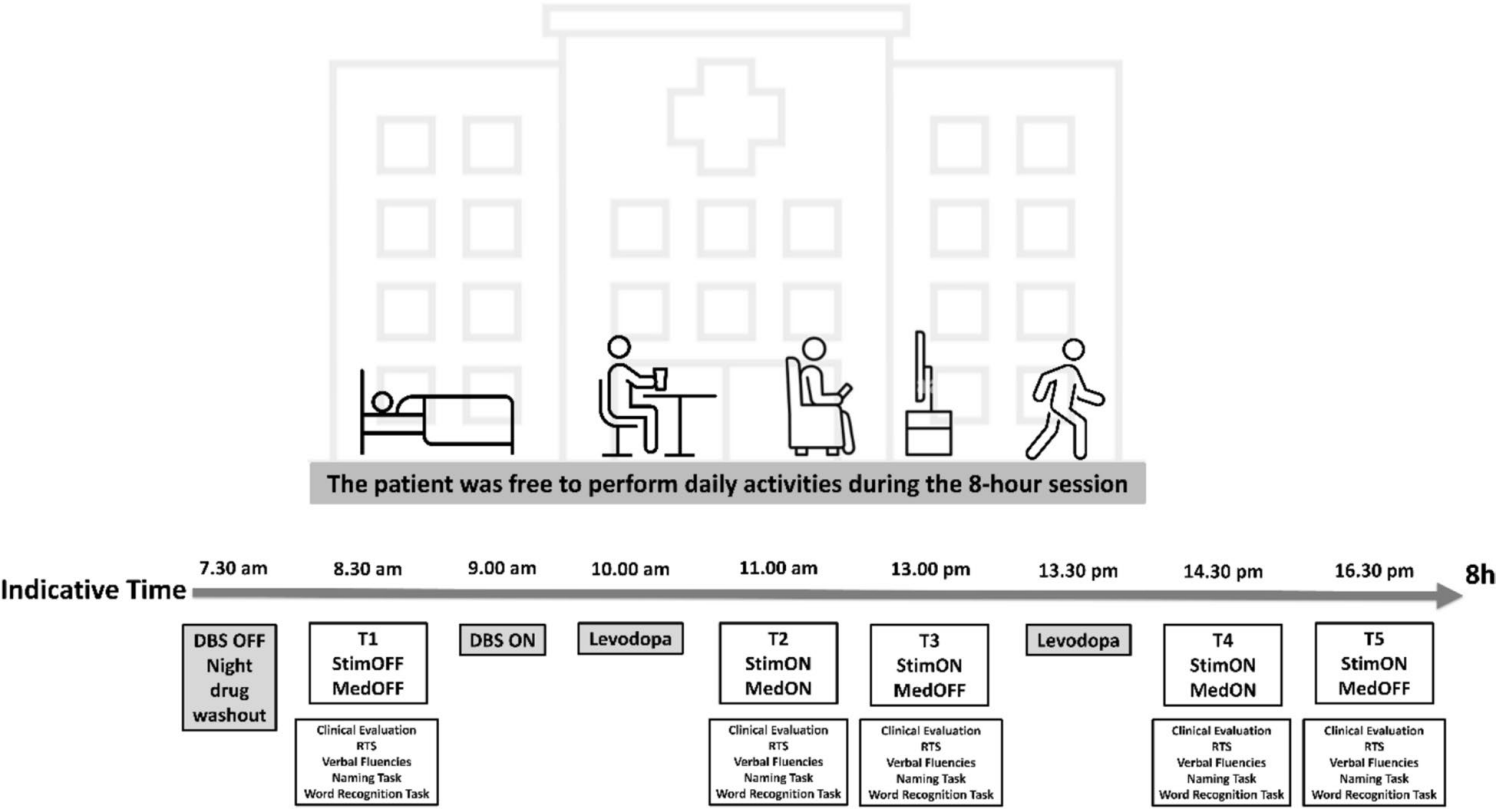
The timeline depicts the 8 h aDBS experimental protocol. Patients were free to engage in daily activities within the hospital between assessments. Cognitive and clinical evaluations were performed at five time points under different stimulation and medication conditions: T1: DBS ‘off’/medication ‘off’ (baseline, after overnight washout); T2 and T4: DBS on/medication ‘on’ (≈60 min post-levodopa intake); T3 and T5: DBS ‘on’/medication ‘off’ (≈60–90 min post-peak dose). Each session included clinical motor evaluation, reaction times task, verbal fluency, naming, and word recognition tasks. aDBS remained active throughout the day, except at T1. DBS: deep brain stimulation; aDBS: adaptive DBS; StimON/OFF: stimulation on/off; MedON/OFF: medication on/off; RTs: reaction times

The patients were prospectively examined 5 days after the DBS surgery according to the aDBS experimental procedure described in Arlotti et al. [15] and Bocci et al [16]. (see Fig. 1 for a schematic overview). The protocol consisted of three evaluation sessions. After 12 h of drug withdrawal, the first session began with a baseline cognitive assessment characterized by both medication and aDBS in the “off” state (T1). Immediately after the basal assessment, aDBS was turned on for the next 8 h. During this simulation period, patients took their first morning medication and underwent a second session of cognitive testing: once in the medication “on” state (peak dose, T2; ≈60 min after medication intake) and once in the medication “off” state (end dose, T3; ≈60–90 min after peak dose). Peak dose and end-dose phases were identified through clinical motor evaluations by experienced neurologists. The same procedure was repeated again later during the 8 h stimulation period, following the second daily medication dose, taken approximately 3 h after the first. They were then retested in the third session under both medication “on” (second peak dose, T4) and medication “off” condition (second end dose, T5) [15, 16].

We used a neuropsychological test battery to assess language—semantic and phonemic verbal fluency (SVF; PVF) tasks and a naming task (Naming) [17, 18], memory—visual-recognition task (Recognition) [18], and attention—simple reaction times task (RTs). In this task, a square appears on the screen at random intervals, and the subject must quickly press the space bar after the stimulus appearance. Median reaction times and omissions are recorded [19]. Parallel versions of all cognitive tasks, including verbal fluency, semantic fluency, and naming, were used at each time point to minimize repetition and learning effects.

In addition, four patients also underwent the same assessment—in terms of tasks adopted and time points addressed—under an 8 h cDBS protocol.

### Statistical analyses

Raw variables were checked for normality by addressing skewness and kurtosis values, which were judged as abnormal if > |1| and |3|, respectively [20].

Accordingly, either parametric or non-parametric tests were employed for assessing the trend in cognitive performances across the five time points under the aDBS protocol. More specifically, a repeated-measure ANOVA was run on PVF scores, while Friedman’s tests were employed when addressing the remaining measures (i.e., RTs as well as SVF, Naming and Recognition scores).

By contrast, to assess the trends in cognitive performances across the five time points under the cDBS protocol, Friedman’s tests were employed for all the outcomes due to the restricted number of patients included (i.e., *N* = 4).

Analyses were run via Jamovi 2.3 (https://www.jamovi.org/) and SPSS 27 (IBM Corp., 2020). The significance threshold was set at *α* = 0.05. Post hoc comparisons were adjusted via Bonferroni’s method within the repeated-measure ANOVA and via Durbin–Conover’s within Friedman’s tests.

## Results

Table 2 summarizes cognitive scores of patients (*N* = 16) undergoing the aDBS and cDBS (*N* = 4) protocol across the five time points.

**Table 2.** Cognitive scores of patients undergoing aDBS and cDBS across the five time points

aDBS						
Measure	T1	T2	T3	T4	T5	*p* value
RTs	467.4 ± 148.2 (311.4–946.7)	462.8 ± 108.6 (295.3–689.3)	521.2 ± 224 (335.4–1312.5)	449.3 ± 93.4 (304.4–645)	385.3 ± 66 (325.8–544.6)	0.110^a^
PVF	11.6 ± 3.8 (5–18)	10.6 ± 3.9 (5–18)	12.3 ± 4.3 (4–21)	11.8 ± 4.8 (5–20)	10.6 ± 4.1 (3–20)	0.214^b^
SVF	15.1 ± 7.9 (3–31)	14.2 ± 6.3 (1–32)	12.8 ± 3.7 (7–21)	13.4 ± 3.6 (5–19)	15.7 ± 4.5 (9–25)	0.419^a^
Naming	19.7 ± 0.6 (18–20)	19.7 ± 0.6 (18–20)	19.3 ± 1.2 (16–20)	19.6 ± 0.6 (18–20)	19.5 ± 0.9 (17–20)	0.654^a^
Recognition	19.4 ± 3.1 (10–23)	19.2 ± 3.7 (10–23)	19.1 ± 4.7 (6–24)	19.4 ± 4 (8–24)	18.4 ± 3.7 (7–22)	0.118^a^
cDBS						
RTs	419.4 ± 84.4 (304.7–507.9)	564.5 ± 234.2 (313.8–806.6)	636 ± 369.8 (324.5–1163.1)	399.1 ± 80.2 (296.6–492.2)	481.7 ± 100.2 (332.8–547.1)	**0.019**^**a**^
PVF	11.5 ± 4.5 (6–17)	9.5 ± 4 (6–15)	9.2 ± 4.6 (6–16)	10.2 ± 1.3 (9–12)	12 ± 2.9 (9–15)	0.399^b^
SVF	11 ± 2.3 (9–13)	10.5 ± 2.4 (9–14)	8.8 ± 5.3 (1–3)	12 ± 4.5 (6–17)	12.5 ± 7.1 (7–23)	0.791^a^
Naming	19.8 ± 0.5 (19–20)	19.2 ± 0.5 (19–20)	19.8 ± 0.5 (19–20)	19.8 ± 0.5 (19–20)	19.8 ± 0.5 (19–20)	0.525^a^
Recognition	22.5 ± 1.3 (21–24)	19 ± 2.4 (16–21)	21 ± 2.4 (18–23)	20.5 ± 2.6 (18–24)	19.8 ± 4 (14–23)	0.055^a^

Values are reported as mean ± standard error of the mean (SEM) (range: minimum–maximum).

aDBS: adaptive deep brain stimulation; cDBS: conventional deep brain stimulation; T1: aDBS “off”–medication “off”; T2: aDBS “on”–medication “on”; T3: aDBS “on”–medication “off”; T4: aDBS “on”–medication “on”; T5: aDBS “on”–medication “off”; RTs: reaction times; PVF: phonemic verbal fluency; SVF: semantic verbal fluency

^a^p value associated with the *χ*^*2*^ statistic within a Friedman’s test

^b^p value associated with the *F* statistic within a repeated-measure ANOVA

In patients undergoing aDBS, no main effects of time were detected on RTs (*χ*^2^(4) = 7.55; *p* = 0.110), PVF scores (*F*(4,60) = 1.50;* p* = 0.214), SVF scores (*χ*^2^(4) = 3.91; *p* = 0.419), Naming scores (*χ*^2^(4) = 2.45; *p* = 0.654), and Recognition scores (*χ*^2^(4) = 7.37; *p* = 0.118).

**Fig. 2.**
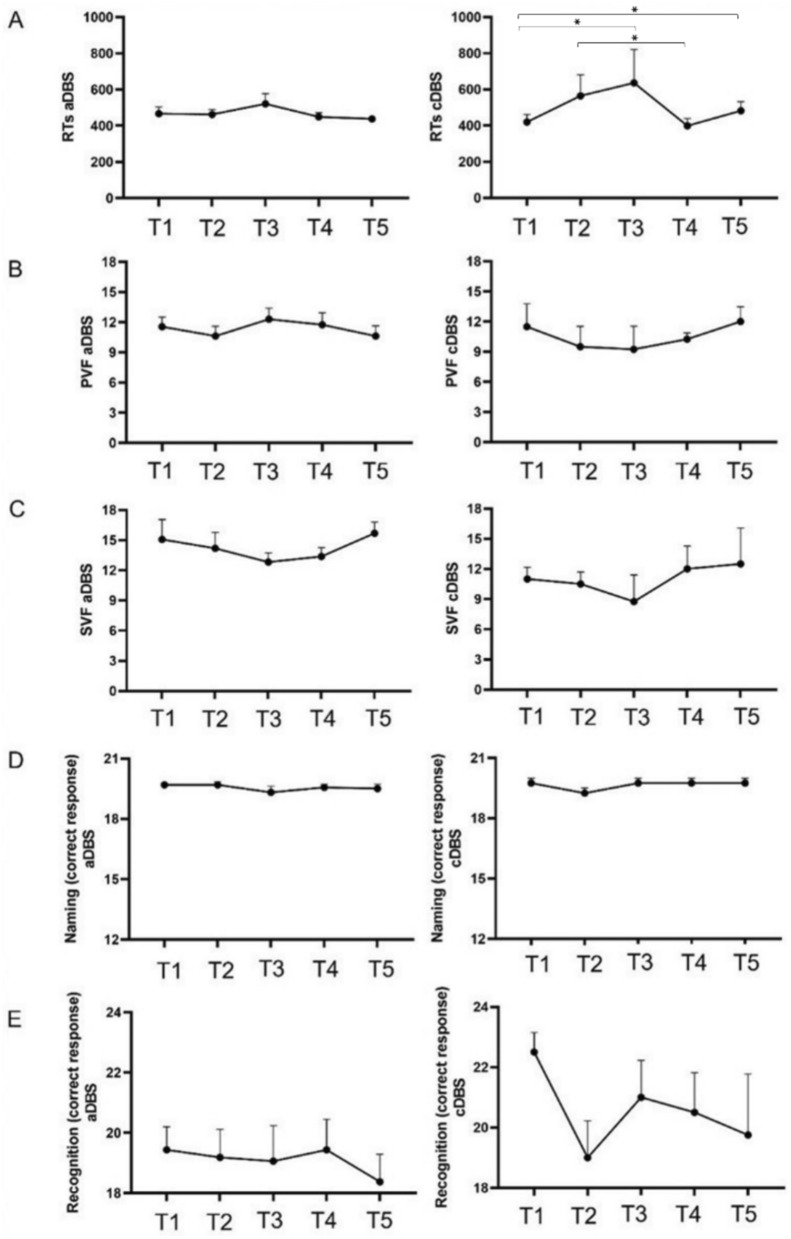
Trends in cognitive score across the five time points, on the left adaptive DBS (aDBS), on the right conventional DBS (cDBS). **A** Reaction times (RTs); (**B**) phonemic verbal fluency (PVF) scores; (**C**) semantic verbal fluency (SVF) scores; (**D**) Naming scores; (**E**) Recognition scores. T1: aDBS “off”–medication “off”; T2: aDBS “on”–medication “on”; T3: aDBS “on”–medication “off”; T4: aDBS “on”–medication “on”; T5: aDBS “on”–medication “off”. Data are expressed as mean ± standard error of the mean (SEM). Higher scores on the y-axis indicate better performances for all cognitive measures except for RTs, where lower scores indicate better performances

A significant main effect of time on RTs was found in patients undergoing cDBS (*χ*^2^(4) = 11.80; *p* = 0.019). Post hoc comparisons by Durbin–Conover revealed that RTs were lower at T1 than at T3 (*p* = 0.011) and T5 (*p* = 0.021), suggesting that RTs worsened and fluctuated during medication “off” status. Considering both cDBS and medication “on” status, we found that RTs were lower at T4 compared to T2 (*p* = 0.002). No significant effects were detected on PVF (*F*(4) = 4.06; *p* = 0.399), SVF (*χ*^2^(4) = 1.70; *p* = 0.791), Naming scores (*χ*^2^(4) = 3.20; *p* =.525), and Recognition scores (*χ*^2^(4) = 9.26; *p* = 0.055). Cognitive scores over times are depicted in Fig. 2.

## Discussion

The present study provides preliminary evidence for the cognitive safety of aDBS of the STN in PD patients. Indeed, the current 8-h protocol resulted in no significant changes in performance on a range of tasks assessing attention, language, and memory throughout the day. These are the cognitive functions known to be most affected by traditional STN-DBS systems [1].

In contrast to the results regarding aDBS, diurnal fluctuations in RTs were observed in patients who underwent cDBS. RTs in PD are influenced by both motor and non-motor components [21] and are a valid and reliable measure of patients’ response to STN-DBS [22]. Previous studies demonstrated that bioelectrical activity in temporal-parietal circuits is involved in motor response in a three-stimulus paradigm [23]. Temporal–parietal circuits are linked to the STN, and a suboptimal response to STN-DBS could reflect a maladaptive activation of these circuits leading to slower RTs [22]. It might be speculated that aDBS invokes plastic changes in the nervous system that outlive the effects of continuous electrical stimulation delivered by cDBS.

Interestingly, patients’ performance on the other tasks assessing language and memory did not significantly change over time under either the aDBS or the cDBS protocol. The results concerning cDBS are overall in line with the current, heterogeneous knowledge regarding the cognitive side effects of STN-DBS: indeed, while several studies reported post-operative cognitive changes, others did not [8]. Verbal performance could be influenced by the frequency of stimulation with better performance with low frequency stimulation and poorer performance when a patient is stimulated with high frequencies [23]; it is, thus, possible that non-motor area of the STN is sensitive to high frequency and that delivering a large amount of current to this area may have an impact on verbal fluency.

Such a stance appears to be supported also by recent evidence suggesting that, in patients implanted with cDBS, higher TEED values are associated with cognitive and behavioral dysfunctions after surgery [9, 10]. Adaptively delivering the current in a closed-loop manner [15, 16] based on patients’ needs and clinical status—only when pathological beta-activity is detected, as in aDBS—reduces overall current and limits spread to non-motor regions of the STN which are functionally connected to cognitive networks. This approach may help mitigate the cognitive side effects of the stimulation. Indeed, it has been hypothesized that current spread to these areas may create an “informational lesion” that disrupts physiological cognitive processing within non-motor circuits, potentially impairing cognitive performance [12]. By minimizing off-target stimulation, aDBS may help preserve the integrity of cognitive networks. Furthermore, Darbin et al, [24], in a monkey study, showed that aDBS is an effective therapeutic approach which requires a lower electrical charge delivery than cDBS for comparable clinical benefits. Our preliminary findings suggest that aDBS may be cognitively safe in our sample, as adapting the stimulation to the needs of the patients did not appear to impair their cognitive performance. This aspect needs to be further investigated in larger samples to corroborate this assumption.

Our study has several limitations. The limited and imbalanced group sizes may have disproportionately influenced the results. In addition, we could not assess cognitive performance in an L-dopa “on” and aDBS “off” condition, limiting insights into the interplay between dopaminergic drugs and DBS. A potential lesional effect from surgery or peri-lesional edema cannot be excluded, highlighting the need for longitudinal studies. Moreover, the small number of cDBS patients prevented a direct comparison with aDBS, warranting future research on the cognitive safety of both techniques. This last stance similarly applies to our analyses on patients undergoing cDBS: the extremely restricted sample size limits the generalizability of our findings in this respect. Finally, as to the present statistical plan, linear/generalized linear mixed models could have been adopted instead of repeated-measure ANOVAs/Friedman’s tests; the former indeed allow for a more flexible modeling of data when compared to the latter. We advocate that future investigations embrace the former statistical framework; in fact, we deemed more reasonable to address the latter due to the limited sample size.

Future research is, therefore, advisable that focuses on comparing the cognitive safety of the two techniques, either via a within- or a between-subject design.

In conclusion, the present report provides promising, preliminary evidence that aDBS to STN is cognitively safe and stable, encouraging further research to confirm that aDBS represents a feasible evolution of traditional continuous DBS.

## Data Availability

The data supporting the findings of this study are available from the corresponding author upon reasonable request due to proprietary restrictions associated with the device under investigation.
